# Consumer Preference for Food Bundles under Cognitive Load: A Grocery Shopping Experiment

**DOI:** 10.3390/foods11070973

**Published:** 2022-03-27

**Authors:** Kathryn A. Carroll, Anya Samek, Lydia Zepeda

**Affiliations:** 1 Department of Nutrition and Family Sciences, University of Central Arkansas, Conway, AR 72035, USA; 2 Rady School of Management, University of California San Diego, La Jolla, CA 92093, USA; asamek@ucsd.edu; 3 Department of Consumer Science, University of Wisconsin-Madison, Madison, WI 53706, USA; lzepeda@wisc.edu

**Keywords:** food choice, consumer choice, food bundling, cognitive load, grocery shopping, field experiment, consumer behavior

## Abstract

Product bundling is a common retail marketing strategy. The bundling of food items has the potential to increase profits in the grocery sector, particularly for fresh produce, which often has lower profit margins. Although prior work suggests consumers prefer bundles because they require less cognitive effort to select, no study has yet experimentally manipulated cognitive load when food bundles are included in the choice set. To test whether bundle preference differs when cognitive resources are constrained, a grocery shopping experiment was conducted with 250 consumers in the midwestern U.S., in a laboratory that featured a grocery store display. Consumers who grocery shopped under cognitive load had a higher odds of selecting a food bundle even when the bundle did not offer a price discount. Results suggest food bundles may be preferred because they require less cognitive effort to process, which could benefit consumers by simplifying the grocery shopping experience. Additional factors found to influence food bundle selection included whether the bundled items were perceived as being complementary and hunger levels. Food bundles could help lessen cognitive effort associated with grocery shopping and may especially appeal to those who do not enjoy food shopping.

## 1. Introduction

The bundling of two or more items offered for sale at a single price is a common retail strategy frequently seen in the marketplace [1,2]. Benefits of displaying product bundles in the food sector exist for both retailers and consumers. From the retail perspective, bundles of food items could help move a greater volume of certain products. Bundling slow-selling items with more popular sellers could also be a potential strategy for both introducing new products and for moving more vulnerable or perishable items, such as fresh produce. Bundling slow-selling produce items together either as a bundle containing one type of product or different products may help increase profits. Identifying factors that influence consumer selection of product bundles also would be helpful information then for food retailers.

Consumers may also benefit from product bundles if the bundle offers a price discount compared to the summed price of the individual items. Although product bundles often feature a price discount, it is possible they may be preferred by consumers for other reasons as well. For example, preference for bundles may be influenced by whether the bundled items are complimentary items that are perceived as fitting well together [3]. Selecting a bundle rather than multiple individual items may also benefit consumers by both simplifying shopping and shortening the amount of time it takes to complete a shopping trip.

Prior work by Harris and Blair [4] and Sharpe and Staelin [5] suggests consumers may prefer bundles because bundle selection requires less cognitive effort—resulting in less information processing and a reduction in consumer search efforts. In addition to cognitive load, cognitive traits, such as one’s need for cognition, have also been suggested as having an influence on the likelihood of bundle selection. Need for cognition can be thought of as an individual’s motivation to process information and their inclination to engage in effortful thinking [6,7,8,9]. Consumers with lower need for cognition have been found to exhibit even greater preference for bundled products [4].

To the knowledge of the authors, no study has pointedly examined whether there is a tendency for consumers to select food bundles when cognitive resources are limited by actually manipulating cognitive load during a non-hypothetical shopping task. If consumers prefer food bundles because they require less cognitive effort, one would expect preference for bundles to increase when shopping under cognitive strain. Consumers with a lower need for cognition would likewise be expected to exhibit even greater preference for food bundles. The influence of price discounts for bundled items when shopping under cognitive load and whether consumers perceive the bundled items to be complementary also warrants investigation. It is possible that additional factors beyond cognitive effort and discounting, such as hunger, whether bundled items are viewed as complimentary, and enjoyment of shopping, may also influence bundle selection.

Having a better understanding as to the cognitive factors influencing bundle selection could be helpful information for grocery retailers and other direct-sales venues, such as farmers’ markets and co-ops. Such venues may wish to utilize product bundling as a marketing strategy designed to increase profits for their operations as well as appeal to consumers. Retail promotion of grocery bundles could be an effective and inexpensive display strategy to implement in retail stores and other food purchasing venues. Bundles may also reduce consumers’ cognitive load by simplifying their shopping experience.

The objectives of this research then are to: (1) determine if consumers who grocery shop under high cognitive load are more likely to select a bundle and if bundles need to be discounted, (2) uncover if consumers with a lower need for cognition exhibit increased bundle selection compared to other consumers, and (3) identify additional factors that could influence grocery bundle selection, including whether consumers perceive the bundled items to be complementary, enjoyment of shopping, hunger, and demographics.

To address these objectives, we conducted an artefactual field experiment with 250 community participants in the midwestern U.S., in a laboratory turned into a grocery store, where both individual food items and food bundles are included in the choice set. We exogenously varied whether or not the displayed bundles in the choice set offered a price discount. Participants completed two food-shopping tasks using the same choice set: once under high cognitive strain (high load) and once under no cognitive strain (no load). Post-experiment measures of need for cognition and other potential factors were collected via questionnaire. Results suggest food bundles may be preferred because they require less cognitive effort to process, which could benefit consumers by simplifying the grocery shopping experience.

## 2. Literature Review

### 2.1. Product Bundling as a Marketing Strategy

While the justification behind consumer preference for bundles remains an area in need of further research, it is a common retail marketing strategy [2]. Likewise, the bundling of complementary items is a common selling technique, where functionally related items are sold together for a single stated price [10]. Bundled products are routinely seen for electronics as well as travel purchases; products may be offered as pure bundles (components only offered for sale as part of the bundle), or mixed bundles (components also offered for sale individually) [11,12]. Complementary product bundling, where products are either functionally related or fit well together, is also a common retail strategy. Prior studies have suggested that consumer preference for bundles may be influenced by whether the bundle combines complementary products [3,13].

In the food sector, bundled items are often seen at fast food establishments and restaurants and may be discounted compared to purchasing the components individually. Grocery stores in the U.S., such as Trader Joe’s, have been known to sell bundles of complimentary items, such as a make-your-own salsa basket, as have farmers’ markets. Amazon, now an online grocery retailer, also offers “grocery value bundles” as an option for shoppers. However, the majority of U.S. grocery stores more commonly employ price bundling (e.g., pick 10 items for USD 10) rather than selling a single bundle containing multiple items.

While some bundles may offer a price savings, at other times, they may be priced higher than if one were to purchase the items individually [10]. Bundling’s simplification of choices has also been found to have a positive effect on consumer purchasing likelihood, which may also appeal to retailers [14].

Grocery stores and direct sales venues, such as farmers’ markets, perhaps have the most to gain from utilizing product bundles as a marketing strategy. Compared to shelf-stable foods, the produce section of a grocery store often offers greater opportunity for higher profit margins [15]. This is especially important, as the grocery industry in the U.S. is noted as having thin profit margins that are frequently less than 3% [16].

Fresh produce items are often highly perishable with a shortened shelf-life compared to most other foods. Produce shrinkage, a term used to describe the amount of produce received by a retailer but not sold, is a common problem. Buzby et al. [17] estimated the average total shrinkage for U.S. produce sections in 2012 was just under 5% of retail sales, amounting to approximately 6 billion pounds each for fresh fruits and vegetables. Although produce shrinkage occurs for a variety of reasons, one way to reduce in-store shrinkage is to ensure perishable items sell before they spoil. Prior work has found that healthy product bundles may even be useful for increasing consumer fruit and vegetable selection [18].

### 2.2. Consumer Benefits of Product Bundling

The most obvious consumer benefit of product bundles would be if the bundle offered a monetary discount compared to the summed price of the individual items. Although some bundles in the U.S. food sector do offer a discount (such as Chili’s© Grill & Bar’s USD “$22 dinner for 2” meal), other benefits beyond price savings may exist. Work by Johnson et al. [19] concluded that a consumer will exhibit more positive evaluations for a bundle rather than individual items largely due to the bundle’s single price. Later Sharpe and Staelin [5] found consumers tended to view bundles as being of increased value and argued that bundling may lessen cognitive effort. The price of the bundle is viewed by the consumer as a single monetary loss as opposed to a series of several losses if the same items were purchased individually. Following the seminal work of Thaler [20] on how individuals account for gains and losses, consumers may be more likely to purchase a bundled product because of its single price. This may especially be the case for those shopping with a set budget, who perhaps are more sensitive to prices (and perceived losses).

Additional benefits of product bundles primarily focus on how consumers cognitively process bundles. Park et al. [21] noted consumers routinely shop for groceries while under mental strain and that grocery shopping itself can be cognitively stressful. Consumers are also faced with a multitude of items when entering the average grocery store: an average of 31,119 different products in 2020 [22]. For shoppers, this plethora of offerings results in having to routinely perform in-store search activities while regularly shopping under time constraints [23]. Other sales venues, such as farmers’ markets, may also be cognitively stressful to shop. At the average farmers’ market, shoppers not only have to deal with multiple sales transactions but also an ever-changing product offering.

Prior work by Guiltinan [24] suggested consumers may prefer bundles because of the value they provide in reducing search efforts—not having to search for and assemble a set of individual selections. Work by Harris and Blair [4] has shown that consumers may prefer bundled choices over individual options, as it has been suggested that bundle selection reduces search efforts and requires less information processing. However, although Harris and Blair propose that bundle preference may be associated with an inclination towards reducing one’s mental strain, cognitive load was not directly manipulated. In addition, food shopping was not investigated, which is a routine decision-making task that most consumers are familiar with. Whether or not consumer preference for bundles is influenced by cognitive strain is an area in need of further research. If consumer demand for bundles increases when faced with a cognitively stressful decision-making task, this would be helpful information for both consumers and retailers.

### 2.3. Cognitive Resources and Decision Making

Cognitive load, a concept initially introduced by Sweller [25], offers one potential explanation as to why consumers may prefer product bundles when food shopping. Several studies in psychology and economics have examined how limiting cognitive resources can impact consumer preference and decision making [26,27,28,29]. Decision making has been shown to differ depending on whether or not cognitive resources are impaired [30,31,32,33].

Cognitive load theory argues that one’s working memory has a limited capacity with which to process information. When this working memory becomes overloaded, the ability to process information may become affected [34,35]. Kahneman [30,31] investigated cognitive load even further, outlining how cognitive load may then impact behavior and decision making, through use of a two-system framework. These two systems are composed of an intuitive, impulsive System 1, where decisions are completed quickly, and a thoughtful reasoning System 2, which is more analytical [31]. Bundle selection may appeal to grocery shoppers who are having to make in-store decisions using System 1 thinking.

Work by Harris and Blair [4] suggested consumers may prefer bundles over individual choices, as bundle selection requires less information processing and reduces search efforts. If selecting a bundle is cognitively easier for consumers when food shopping, then bundle selection should increase when shopping under cognitive load. However, so far, no study has directly manipulated cognitive load while employing a food decision-making task containing food bundles. In addition, Harris and Blair [4] used a hypothetical decision scenario (purchasing stereo equipment), which may be a decision consumers do not routinely make. Whether Harris and Blair’s initial finding holds when consumers are making more routine, non-hypothetical decisions, such as food shopping, is an area in need of further research.

Offering product bundles for sale could benefit consumers if bundle selection provided a means through which to lessen cognitive processing while grocery shopping. Consumers then may experience an increase in utility, created when bundles are offered, which can be observed through their preference for bundles. Using cognitive load theory, we define the total basket of food items that a consumer selects from a given set of food choices. This total basket of items may include individual food items, denoted by *f*, and product bundles, denoted by *b,* such that $\left(f,\;b\right)\in {\mathbb{R}}_{+}^{L}$ is the consumer’s choice set. The consumer is constrained by their household income *w* so that the shopper’s grocery food budget set is defined as $B\left(\;w\right)=\left\{\left(f,\;b\right)\in {\mathbb{R}}_{+}^{L}\;:p_{f,\;b}\leq w\right\}$. The consumer’s utility maximization problem then is:

(1) $$\left(f,\;b\right)=argmax_{\left(f,\;b\right)\in B\left(w\right)}U\left(f,b\right)$$

When consumers are faced with making a decision under cognitive load, selecting a product bundle would be expected to move the consumer to a new level of utility *U^k^* with the basket of food items selected being $\left(f^{*},b^{*}\right)$. Demand for individual food items and product bundles then are expected to be $f^{*}\leq f$, and b* >b, where *k* = 1…*n* for each of the food-shopping task treatments.

It is likewise anticipated that when bundles are discounted, consumers may shift to a different level for utility *U^k^* with basket of food items $\left(f^{**},b^{**}\right)$, where now demand for f^**^ ≤ f^*^, and $b^{**}\geq b$^*^. These demand effects may be even greater for consumers with lower motivation to process information.

In the absence of cognitive load or a discount on bundles, the consumer remains at the original utility level *U^0^* with basket of food items (f,b). Prices $p_{f,\;b}$ are held constant with the exception of treatments featuring discounted bundles, as the study is conducted during one time-period only. For comparison purposes, each participant will shop under a given, identically set B(w).

If bundle selection requires less cognitive effort, it is expected that:

**H1:**  *Consumers will be more likely to select food bundles when shopping under high cognitive load regardless of whether the bundles are discounted.*

As previously discussed, offering a price discount for a product bundle is a common retail strategy. Therefore, it is also hypothesized that:

**H2:**  *Consumers will be more likely to select food bundles if they offer a price discount compared to when food bundles are not discounted.*

However, if discounting bundles does not significantly influence the likelihood of bundle selection, this may suggest that price discounts may not need to be offered by food retailers. Bundles may provide consumers with increased utility separate from any perceived price savings. Whether or not there is a significant interaction between high cognitive load and discounted bundles is indeterminate.

### 2.4. Individual Characteristics and Situational Factors

In addition to cognitive load, an individual’s cognitive traits have also been found to be related to decision-making outcomes [36]. Several measures of cognitive traits exist. One such measure is the Need for Cognition Scale (NCS) developed by Cacioppo et al. [6]. The NCS measures an individual’s self-reported motivation to process information and their inclination towards engaging in effortful thinking. Another measure is the Cognitive Reflection Test (CRT), which is designed to measure an individual’s inclination towards reflecting on their initial “gut” decision or response using observed behavior [36]. Additional measures of cognitive traits include IQ tests, such as standardized test scores.

While both the NCS and the CRT measure cognitive traits, in the context of examining consumer preference for bundles, measuring one’s motivation to process information is perhaps more relevant. Harris and Blair [4] previously concluded that individuals with lower NCS scores were more likely to select bundles when faced with a hypothetical choice task. However, whether individuals with lower NCS scores are more likely to select bundles when grocery shopping, to the knowledge of the authors, has not yet been examined. It could be that when food shopping, consumers with less motivation to process information might find bundles more appealing regardless of whether the bundle is discounted.

Understanding how consumers with lower NCS scores are influenced by cognitive load, and how this in turn may impact preference for bundles would be helpful information for food retailers looking to better appeal to consumers. Therefore, it is hypothesized that:

**H3:**  *Consumers with lower need for cognition will be more likely to select food bundles compared to other consumers, across treatments.*

**H4:**  *An interaction between lower need for cognition and high cognitive load is further expected to increase the likelihood of bundle selection.*

Evidence of such an interaction would further indicate that bundle preference may be driven in part by bundles requiring less cognitive effort to process.

Situational factors, such as enjoyment of food shopping and hunger, may also significantly influence the likelihood of bundle selection. Prior work by Cheung et al. [37] suggested that in general, increased hunger levels may be associated with more impulsive choices. Such impulsive choices result in decisions that are quickly made, which may align with bundle selection, as bundle selection is quicker than selecting individual items. It is possible that hunger may also impose cognitive load. Those who do not enjoy food shopping may likewise prefer bundles, as bundle selection may reduce the overall number of food-shopping decisions that the individual needs to make. Therefore:

**H5:**  *Additional factors, such as a lack of enjoyment of food shopping, increased hunger, whether the bundled items are perceived as complimentary, and demographics, will significantly influence the likelihood of bundle selection.*

Whether or not there are significant interactions between these additional factors and the main effects of discounted bundles and high cognitive load is indeterminate.

## 3. Materials and Methods

In order to explore whether bundle selection is influenced by cognitive load and offering a price discount, we conducted an artefactual field experiment that featured non-hypothetical food-shopping tasks. Although standard laboratory experiments and artefactual field experiments are similar, an artefactual field experiment differs in that it features participants from the “market of interest” [38,39]; in this case, community participants in the midwestern U.S. who were grocery store shoppers. The experiment was conducted at a university in the midwestern U.S. in 2016. Food-shopping tasks were employed, as prior studies have suggested grocery shopping is cognitively stressful [21,23]. Food shopping is also a common decision-making task that should be familiar to a majority of consumers.

### 3.1. Participant Recruitment and Sample

We recruited 250 participants to complete a single 60 min session, with an average attendance of 9–16 participants per session. Participants were recruited using advertisements featured on online community boards and through postings at area community centers, public libraries, and at local grocery stores. To avoid any potential sample selection bias, recruitment materials referred to the experiment as only a “consumer study”. Participants were prescreened before being invited to a study session. Individuals interested in participating in the study were excluded if: (1) they were not 18, (2) they had not been a U.S. resident for at least 5 years, (3) they had not grocery shopped in the past two months, (4) they were currently full-time undergraduate students, and/or (5) they indicated a food allergy. Our final sample of 250 participants was on average 31 years old (SD = 11.21), 33% male (SD = 47%), and 13% had children under 18 in the household (SD = 33%). In addition, 31% of our sample identified as non-Caucasian (SD = 46%), and the average household income reported was USD 65,300 (SD = USD 47,500). None of the participants in our sample were full-time undergraduate students.

### 3.2. Grocery Shopping Display

The laboratory was converted to mimic a small grocery store featuring 6 different product bundles and 30 different individual food items. These food items consisted of 10 fruit and vegetable items, 10 junk food/snack items, and 10 protein/dairy/grain items. The 6 food bundles consisted of 5 items each; the bundled items could also be purchased individually. This is commonly referred to as mixed bundling: when items that are bundled together can also be purchased individually [12]. These 30 different foods were in quantities and sizes that were appropriate for retail pricing at USD 1. This allowed for the food items to be more easily compared. To ensure that the pricing in our study accurately reflected the marketplace, average retail prices from three local grocery stores were collected weekly throughout the study and ranged between USD 0.89 and USD 1.08 for each item.

A commercial display cooler, freezer, and shelving was also used in the laboratory to help mimic an actual grocery store shopping environment. This also allowed participants to view the actual food items and bundles that they would be selecting, which prior work by Shiv and Fedorikhin [40] suggested is an important aspect of a food-choice study.

### 3.3. Pretest of Food Bundles and Items

All of the individual food items as well as the food bundles were pretested using a focus group of 22 shoppers from an area grocery store. These pretest shoppers were 40.9% male and on average were 42.5 years old (SD = 33.2). Shoppers participating in the pretest focus group were asked to indicate their preference for several different food bundles and individual food items. They were also asked to share their thoughts in general on the various foods and food bundles displayed. Food items and bundles that were not rated well by the focus group participants were ultimately removed from the choice set. A total of 12 different bundles were pretested, and the 6 highest-rated bundles were included in the final study design. Table 1 displays the final 6 food bundles.

### 3.4. Experimental Procedure

Participants were provided with the study purpose and protocol and provided their signed informed consent to participate, when they arrived for their study session. The University of Wisconsin–Madison’s Institutional Review Board for research on human subjects approved the study protocol. Participants received a show-up payment of USD 5 plus the food items and/or food bundles selected during the food-shopping tasks. Participants could also earn up to an additional USD 8 dependent upon their performance during the study session.

**Figure 1:**
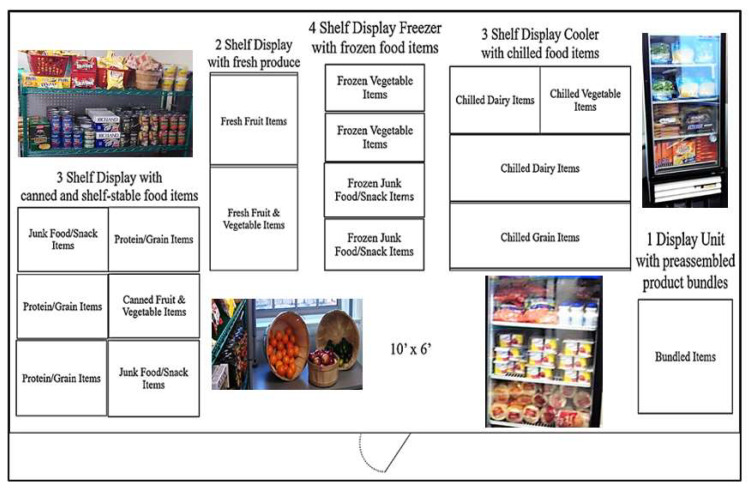
Display layout for the grocery shopping experiment.

Each session began with participants first practicing each of the different types of tasks featured in the experiment. Participants were next asked to complete two food-shopping tasks by visiting the grocery shopping area privately and were given a USD 10 budget to use in each task. Participants were not allowed to use their own personal funds and were instructed to use the full USD 10 for each task, as any unused funds would be forfeited. The food bundles were displayed together towards the entrance of the grocery display, as bundled items can often be found at the entrance of retail stores. The layout of the grocery shopping display can be viewed in Figure 1.

After visiting the grocery shopping area, participants next made their food selections on a computer, with a five-minute break between the two food-shopping tasks. Any food items and/or bundles that were selected were either given to the participant at the end of the session, or if requested, arrangements were made for pickup at a later time. Participants next completed eight arithmetic questions after a second five-minute break. These arithmetic questions were similar to those used by Deck and Jahedi [29] in order to check whether participants were successfully under cognitive load. These arithmetic questions consisted of multiplying *m_1_* by *m_2_*, where integer *m_1_* ~ *U* (13…19) and integer *m_2_ ~ U* (5…9) [29].

Lastly, the session concluded with a post-experiment questionnaire that included the 18-item short form of Cacioppo et al.’s [6] Need for Cognition Scale (NCS), which Harris and Blair [4] earlier utilized in their study on bundle preference. This allows the results of this study to be more cleanly compared with Harris and Blair’s earlier work. To explore additional factors that may be related to bundle preference, the questionnaire asked whether participants felt the items in the bundle went well together. Harlam et al. [3] concluded that preference for bundles may be influenced by whether the bundled items are complimentary items. Participants were also asked questions about their hunger, enjoyment of food shopping, whether they felt the bundled items were complementary and dietary and standard demographics.

After the post-experiment questionnaire, one of the two food tasks were randomly selected, and participants received the food items and/or food bundles they had selected in that task as well as their final payments. Further details as to the order of the study tasks for the grocery shopping experiment can be viewed in the Supplementary Materials.

### 3.5. Experimental Design

Participants were randomly assigned to view one of the two different grocery displays for the duration of their session. The bundles display consisted of 6 different preassembled bundles plus 30 individual items. The individual items consisted of 10 items from each of the following food groups: fruit and vegetable items, junk food and snack items, and protein, dairy, grain items. Items from each of these food groups were included in order to be representative of the various types of foods commonly sold in a grocery store setting. The bundles featured primarily fruit and vegetable items and stated “*5 items for $5*” USD. Each of the six bundles contained at least two fresh produce items, to explore whether consumers would be willing to select bundles that contained more perishable items. The Discounted Bundles display differed from the Bundles display only in that the bundles were discounted 20% and thus priced at “*5 items for $4*” USD.

To explore the effect of cognitive load on bundle selection, each participant completed the same shopping task using the same display version twice: once under high cognitive load (HL) and once under no cognitive load (NL). Within their randomly assigned display, the order in which participants completed the cognitive load conditions was also randomly assigned to address any possible order effects. Participants took a five-minute break between the two shopping tasks, which was used to help eliminate any possible spillover effects between the two tasks caused by the prior task’s cognitive load condition.

Following Deck and Jahedi [29], participants viewed and were asked to memorize a 7-digit number before completing the task under high cognitive load. After the task, they were then asked to recall the number. This resulted in four different treatments for the grocery shopping experiment: 2 displays (bundles vs. discounted bundles) by 2 cognitive load conditions (high load vs. no load). Each participant completed two treatments. The experimental design then allowed for both between-subjects comparisons (between displays) and within-subjects comparisons (between cognitive load conditions). The treatments for the grocery shopping experiment can be viewed in Table 2.

The ability to compare the two displays between-subjects allows for a better understanding of the role of the discount on bundle preference. The ability to compare bundle selection within subjects allows for a better understanding of how an individual’s decisions might change when cognitive resources are limited. Another advantage of comparing within subjects is it allows for the ability to conduct pairwise comparisons, thus helping control for potential individual differences while maximizing sample size. A total of 126 participants shopped under the bundles display, and 124 participants shopped under the discounted bundles display.

### 3.6. Cognitive Load Manipulation Checks

Participant recall accuracy for the displayed 7-digit number was assessed for the two high load treatments: T1-HL (bundles–high load) and T2-HL (discounted bundles–high load). Across both treatments, recall accuracy was close to 90%, with no significant differences observed between treatments or between presentation orders. Differences in arithmetic performance when under cognitive load were also examined, following Deck and Jahedi [29]. If participant recall performance of the 7-digit number is significantly worse under high cognitive load, then it would indicate that high cognitive load was successfully manipulated. Participants overall were on average 9% less accurate (*p* ≤ 0.001) when solving arithmetic problems under high cognitive load. No significant differences were observed in arithmetic accuracy between treatments using non-parametric Wilcoxon signed-rank and rank-sum tests as appropriate. Lastly, no significant order effects were observed.

### 3.7. Empirical Model 

To determine the effect of cognitive load and discounted bundles on bundle selection, differences in the percentage of participants selecting one or more bundles were compared both within subjects and between subjects. Within-subjects comparisons include T1-HL (bundles–high load) versus T1-NL (bundles–no load) and T2-HL (discounted bundles–high load) versus T2-NL (discounted bundles–no load). Between-subjects comparisons include T1-HL versus T2-HL, T1-NL versus T2-NL, T1-HL versus T2-NL, and T1-NL versus T2-HL.

Differences in the percentage of participants selecting one or more bundles was also examined between participants with lower need for cognition compared to those with moderate to higher need for cognition, both within and between treatments. When comparing treatments within participants, non-parametric Wilcoxon signed-rank tests for matched pairs were used. Likewise, when comparing treatments between participants, non-parametric Wilcoxon rank-sum two-sample tests were used.

In addition to examining differences in the percentage of participants selecting bundles between treatments and NCS scores, a random effects binary logit model was used to model the effects of the grocery display and cognitive load as well as additional explanatory variables on the likelihood of a consumer purchasing any food bundle. Parameter estimates obtained from the model were next used to calculate odds ratios in order to examine the odds of a consumer selecting any food bundle for each variable.

A binary logit was determined to be appropriate for the data, as the dependent variable of interest was relatively evenly split: approximately 63% of participants selected any bundle during the food-shopping tasks. Additionally, a random effects model was employed, as each individual completed two food-shopping tasks. Since this resulted in multiple observations from the same individual, the typical assumption of independence of responses is inappropriate. Using a random effects model allows for observations from each individual to be treated as a panel, controlling for any unobserved heterogeneity.

Following Conaway [41] and Rodriguez and Elo [42], we assumed that conditional of random effects δ*_i_*, the observations have independent Bernoulli distributions, with probabilities defined as:

*g (p_ij_) =* Pr (*A_ij_* = 1 | **X**, **Q**, δ*_i_*) = *F* (η + δ*_i_*) (2)

where *i =* 1,*…*., *n* consumers, and *j =* 1,*….*, *n_i_* observations (food tasks). The dependent variable *A_ij_* = 1 if any bundle was selected during the food choice task and 0 otherwise. Vector **X** consists of treatment dummy variables for cognitive load and discounted bundles: *HighCognitiveLoad* and *DiscountedBundle,* which are 1 if the treatment featured the corresponding condition and 0 otherwise. Vector **Q** consists of additional explanatory variables collected from the post-experiment questionnaire as well as any potential interactions between model covariates *k*. These include the dummy variables *LowerNCSScore, ComplimentaryItems, Hungry, DoesNotEnjoyFoodShopping, PlannedToPurchaseSoon, FollowingSpecialDiet, ChildrenUnder18, NonCaucasian,* and *Male*. *Age* in years and *HouseholdIncome10K* are also included. As both need for cognition scores (NCS) and shopping enjoyment were collected as ordinal variables, dummy variables for our levels of interest for both (*LowerNCSScore* and *DoesNotEnjoyFoodShopping*) were included in the model in order to test hypotheses H3 and H5 and examine their effect on the odds of selecting any food bundle. A description of model variables with the measurements for each variable can be viewed in Table 3.

The standard logistic distribution with c.d.f., *F*, from Rodriguez and Elo [37], is defined as *F* (η) = e^η^/(1 + e^η^). The resulting inverse transformation of *F* results in the logit, which can be expressed as:

logit (*p_ij_*) = log (*p_ij_*/(1 − *p_ij_*)) = η + δ*_i_* + ɛ*_ij_* (3)

with η = *β_0_* + ∑ *β_k_* **X** + ∑ *β_k_* **Q**. ɛ_i*i*_ is defined as the error term with a zero mean across consumers and is i.i.d (independent and identically distributed) independently of δ*_i_*. Equation (3) represents the random effects binary logit model and is estimated via maximum likelihood in Stata 17.0.

It is important to note a potential concern of including interaction effects in nonlinear models: the possible influence of unobserved heterogeneity on the estimated interaction effect(s) [43]. However recent arguments have suggested that a benefit of the logit model lies in the interpretation of the dependent variable, which, here, is the probability that a participant selected any bundle. This probability is a function of the information available (the variables included in the model). As long as care is taken in interpreting the dependent variable in this manner, it can be appropriate to include interaction terms in nonlinear models [44,45].

Karaca-Mandic et al. [44] noteD that using odds ratios is the only way to directly interpret the coefficient estimates of the random effects logit model. Buis [43] also discussed the benefits of interpreting interactions in nonlinear models as “multiplicative effects” (odds ratios). Therefore, the model estimated in Equation (3) above incorporates interaction effects in order to gain a better understanding of bundle selection, with odds ratios next computed from the estimates.

## 4. Results

### 4.1. Comparison Statistics

The percentage of participants selecting bundles by treatment and presentation order is presented in Table 4. The highest percentage of participants selecting any bundle (78.23%) is observed in treatment T2-NL (discounted bundles–no load). The second highest percentage of participants selecting any bundle (70.63%) is observed in treatment T1-HL (bundles–high load). In contrast, the lowest percentage of participants selecting any bundle (46.83%) is observed in treatment T1-NL (bundles–no load). As each participant completed two treatments (two food-shopping tasks), differences in the percentage of bundles selected between treatment presentation order were also examined to check for possible order effects.

No significant differences were observed within each treatment, using non-parametric Wilcoxon signed-rank tests for matched pairs. Shapiro–Wilk tests for normality were performed earlier on the percentage of participants selecting any bundle. The results indicated the rejection of normality at better than the 1% level, indicating that the use of non-parametric tests was appropriate.

Differences in the percentage of participants selecting any bundle was compared between treatments in Table 5. As each participant completed two treatments (two shopping tasks), treatment comparisons within participants are first discussed. For participants randomly assigned to shop the bundles display (T1), shopping under high cognitive load (T1-HL) resulted in an average of 23.81% more participants (*p* ≤ 0.001) selecting any bundle compared to shopping under no cognitive load (T1-NL). For participants randomly assigned to shop the discounted bundles display (T2), shopping under high cognitive load appears to have the opposite effect. In the absence of cognitive load (T2-NL) resulted in an average of 18.55% more participants (*p* = 0.002) selecting any bundle compared to shopping under high cognitive load (T2-HL).

Treatment comparisons between participants are next discussed. When shopping under high cognitive load, 10.96% more participants (*p* = 0.070) selected any bundle when the bundles were not discounted. However, when shopping without high cognitive load, discounted bundles were preferred: 31.4% more participants selected any bundle (*p* ≤ 0.001).

**Figure 2:**
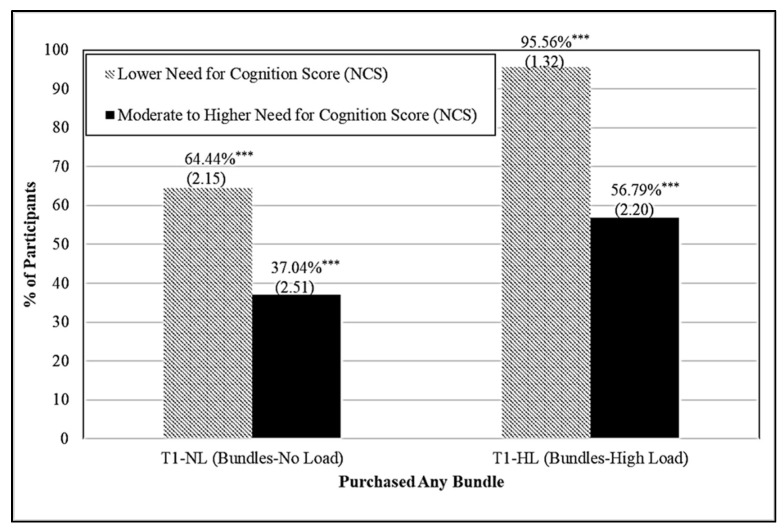
Food Bundle Selection by Treatment (when Bundles were Displayed) and Need for Cognition Score (NCS). NOTE: Standard errors in parentheses. Differences within treatments between need for cognition scores (NCS) are significant at the 1% *** level.

Differences in bundle selection by participants’ self-reported need for cognition (NCS) are next discussed. Figure 2 graphically shows the percentage of participants selecting any bundle, who were randomly assigned to shop under the bundles display (treatments T1-NL and T1-HL), by need for cognition. Participants with a lower need for cognition were more likely to purchase any bundle (*p* ≤ 0.001) both when shopping in the absence of cognitive load (27.4% more participants) and when under cognitive load (38.77% more participants).

**Figure 3:**
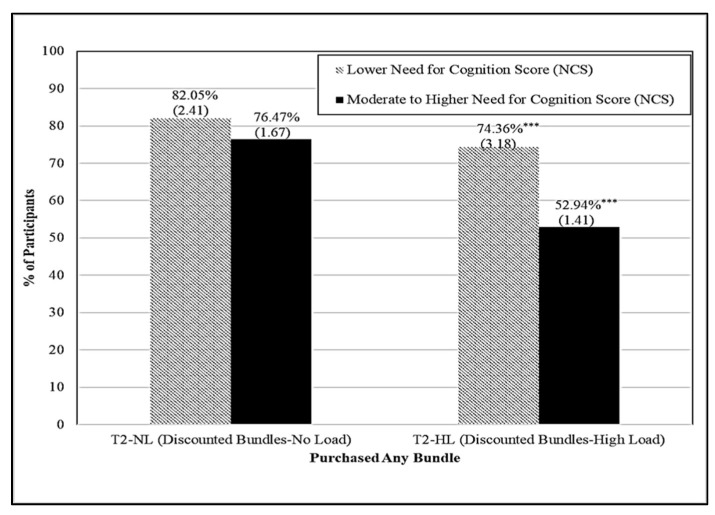
Bundle Selection by Treatment (when Discount Bundles were Displayed) and Need for Cognition Score (NCS). NOTE: Standard errors in parentheses. Differences within treatments between need for cognition scores (NCS) are significant at the 1% *** level if noted.

Figure 3 graphically shows the percentage of participants selecting any bundle, who were randomly assigned to shop under the discounted bundles display (treatments T2-NL and T2-HL), by need for cognition. When shopping under high cognitive load, participants with a lower need for cognition were more likely to purchase any bundle (*p* ≤ 0.001) when bundles were discounted 20% (21.42% more participants). However, no significant differences in bundle selection were observed by need for cognition when participants were not under cognitive load and when discounted bundles were displayed. 

### 4.2. Odds Ratios

The estimated coefficients of the random effects binary logit model were next used to calculate odds ratios and can be viewed in Table 6. To check for model misspecification, a similarly specified random effects probit model was also estimated. The resulting coefficient estimates between the random effects logit and probit were similar, with parameter significance the same. Akaike’s and Schwarz’s Bayesian information criteria (AIC and BIC) were compared postestimation between the two models. Both the AIC and the BIC were lower for the random effects logit, indicating a slightly better fit for the collected data. As a robustness check, three alternative model specifications were also estimated. These three alternative specifications consisted of the model presented in Table 6, with the following variables removed: (1) interactions, (2) demographic variables, and (3) both interactions and demographics. These series of robustness checks indicated that the addition and/or removal of these variables did not influence the significance of the main effects presented in Table 6. Robust standard errors are also reported to correct for any possible heteroskedasticity in the error structure.

**Figure 4:**
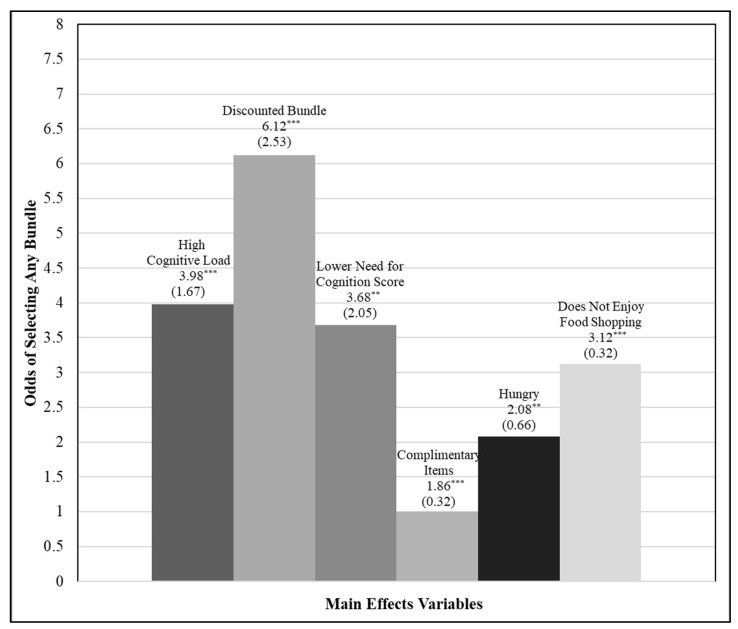
Odds of Selecting Any Bundle, by Main Effects Variables. NOTE: Robust standard errors in parentheses. Odds ratios are significant at the 5% ** or 1% *** level if noted.

The odds ratio can be generally interpreted as the number of participants who select any bundle for every participant who does not select a bundle. Odds ratios for the main effects included in the model are examined first. Figure 4 graphically displays the odds of selecting any bundle, by the main effects included in the model. For participants shopping under *HighCognitiveLoad*, the odds of selecting any bundle is 3.98 times higher than when shopping in the absence of cognitive load. This finding in part supports hypothesis H1 and suggests that consumers are more likely to select a bundle when shopping under cognitive strain.

As hypothesized in H2, consumers are more likely to select a food bundle when the bundle offers a price discount. When the grocery display featured discounted bundles (*DiscountedBundle*), the odds of selecting any bundle are 6.12 times higher than when a price discount is not offered on bundles. Participants with a *LowerNCSscore* were 3.68 times more likely to select any bundle compared to participants with moderate to higher NCS scores. This finding was in line with earlier expectations under hypothesis H3 that participants who are less motivated to process information would be more likely to select any bundle.

Statistically significant interactions are observed between *HighCognitiveLoad* and *DiscountedBundle, LowerNCSscore*, and *Hungry*. When shopping under high cognitive load, participants with a lower NCS score and participants who are *Hungry* are more likely to select any bundle. This significant interaction suggests that for these participants, the main effects of *HighCognitiveLoad, LowerNCSscore,* and *Hungry* have a stronger effect on the probability of bundle selection. The significant interaction observed between high cognitive load and lower motivation to process information supports hypothesis H4, indicating that preference for bundles may in part be due to cognitive ease associated with bundle selection.

Interestingly, the interaction between shopping under high cognitive load when bundles were discounted is estimated to significantly weaken the main effects of *HighCognitiveLoad* and *DiscountedBundle* on the probability of selecting any bundle. This finding was an unexpected result, and although further research is warranted, it may suggest that the price discount moderates the effect of cognitive load—having to mentally account for a price discount while under cognitive strain may negate any perceived benefit offered by the discounted food bundle.

As hypothesized under H5, several additional factors were found to significantly influence bundle selection. Participants who indicated that they were “very” or “extremely” *Hungry* while completing the grocery shopping experiment were 2.08 times more likely to select a bundle compared to participants with lower levels of hunger. One’s enjoyment of food shopping was also found to have a significant effect on likelihood of bundle selection: participants who indicated they did not enjoy food shopping (*DoesNotEnjoyFoodShopping*) were 3.12 times more likely to select any bundle.

When participants considered the items in the displayed bundles to be complimentary (*ComplimentaryItems*), they were 1.86 times more likely to select a bundle compared to those who did not “agree” or “strongly agree” that the items in the bundles went well together. Lastly, as viewed in Table 6, although not hypothesized, both *PlannedToPurchaseSoon* and *FollowingSpecialDiet* significantly decreased the probability of bundle selection. None of the demographic variables included in the model had a significant main effect on the probability of selecting any bundle during the food-shopping task.

## 5. Discussion and Conclusions

The findings uncovered here align with Harris and Blair’s [4] earlier suggestion that preference for bundles may be partially attributed to bundle selection requiring less information processing. Our results indicate that the odds of selecting any bundle was 3.98 times higher for participants shopping under high cognitive load compared to those shopping in the absence of load, significant at the 1% level. This finding also aligns with prior work by Guiltinan [24], who had earlier suggested that bundle preference may be associated with a desire to reduce search efforts and thus simplifies the shopping experience.

A significantly greater percentage of participants selected a food bundle when shopping under high cognitive load even though there was no price savings associated with bundle selection. This effect was even greater for consumers with less motivation to process information, who were even more likely to select any food bundle when shopping under high load. We find that while consumers are more likely to select a food bundle when the bundle is discounted, bundles did not necessarily need to be discounted to appeal to participants in our sample. This finding aligns with prior work by Sharpe and Staelin [5], which argued that bundling may lessen cognitive effort as well as work by Thaler [20] that concluded bundles may be preferred by consumers because of the single stated price of the bundle.

Interestingly, participants with a lower need for cognition were more likely to purchase a bundle regardless of cognitive load condition, significant at the 1% level. This finding supports the earlier conclusions of Harris and Blair [4], who likewise found that consumers with lower need for cognition exhibited greater preference for product bundles. This finding also suggests that while an individual’s cognitive traits are related to decision-making outcomes, as Frederick [36] earlier concluded, such traits may also be related to preference for bundled products. Consumers with less motivation to process information then may find bundles more appealing due to the reduction in search costs that bundle selection provides. Our findings also suggest consumers may prefer food bundles because they require less cognitive effort to process and thus potentially simplify the shopping task. Retailers may find that price discounts for food bundles may not be necessary, as bundles may enhance customer convenience by reducing cognitive load. Bundling may also be particularly useful for retailers needing to move more perishable fresh produce items.

An interesting interaction is observed between high cognitive load and when product bundles offer a price discount, which weakens the main effects of both of these variables on the odds of selecting any bundle. It may be that when faced with cognitive load while grocery shopping, an in-store price discount on food bundles negates the cognitive ease of selecting a bundle when the consumer is shopping under a budget constraint. Another possibility is that discounting bundles, in the experimental design of the study, served as an impediment to completing the task quickly under cognitive load. Whether or not in-store price discounts add cognitive stain to the task of grocery shopping is an area in need of further research. The results presented here suggest that price discounts on food bundles may not be necessary in order to meet the needs of consumers.

Additional factors beyond cognitive load and price discounting were also found to influence bundle selection. Consumers who perceived the bundled items as being complimentary were more likely to select a bundle. This finding has interesting implications for retailers and purchasing venues as they consider the types of food items to bundle. Retailers may want to consider whether the bundled food items go well together or whether they may comprise a meal. This finding also aligns with earlier work that suggested increased preference for bundles may be influenced by whether the bundled items were complementary products [3,13].

Additional individual factors, including a lack of enjoyment of food shopping and increased hunger levels, were also found to significantly influence bundle selection. Participants who indicated experiencing increased hunger levels while they shopped were 2.08 times more likely to select a bundle than those with lower hunger levels. As prior work by Cheung et al. [37] suggested, increased hunger levels may be associated with more impulsive choices and as impulsive choices often result in decisions that are quickly made, bundle selection may appeal to hungry consumers looking to shorten their time spent grocery shopping. This could be particular helpful for grocery stores that cater to shoppers who are shopping after work and are looking for items to prepare for their evening meal. Displaying food bundles in-store may be one way to meet the needs of such consumers.

We also find that those who indicated they did not enjoy food shopping were 3.12 times more likely to select any bundle, perhaps in an effort to shorten the time it took for them to grocery shop. Bundles then may also appeal to consumers who do not enjoy food shopping, which for most individuals is a necessary household task that must be performed. In particular the results here suggest that male shoppers who do not like to grocery shop may perhaps be more likely to select a bundle when food shopping. Retailers then could explore offering bundles that contain food items that might be particularly appealing to this segment of consumers.

In conclusion, the results of this study suggest that food bundles may be preferred by consumers because they require less cognitive effort to process. This in turn could benefit grocery shoppers by simplifying the overall shopping experience. Food bundles may even appeal to those consumers who do not particular enjoy food shopping due to the increased ease of selection associated with choosing a bundled product.

### 5.1. Limitations

A limitation of the study is that it was conducted in a laboratory setting; further work should explore the feasibility of bundle selection in actual retail environments. The experimental design of the study also asked participants to spend their entire USD 10 while they shopped. All of the individual food items featured in the study were also priced at USD 1. We recognize that in an actual retail environment, consumers face a much wider range of prices and are not necessarily having to exhaust their shopping budget. This study also used a grocery display that allowed participants to view the bundles and individual items all at once. It is possible that the in-store location of bundle displays could also impact the likelihood of bundle selection. It is also possible that bundle selection may reflect the complexity of the bundled products. Shopping in a grocery store would also mean being exposed to even more items, which increases complexity and cognitive load. Therefore, a limitation of our experimental design is that it is a very conservative estimate of the preference for bundling under cognitive load. In addition, this study used a between and within-subjects design, which we note may also be a potential limitation. A concern with within-subjects designs is their potential to induce demand effects if the research question becomes clear to participants. However, we failed to find any significant effects of the sequence of the food-shopping task, which suggests such demand effects likely did not occur. Lastly, limitations of our participant sample include a predominately female sample that is relatively young (31 years old) and who are residents of the midwestern U.S.

### 5.2. Retail Suggestions and Further Research

Future work is needed on the implementation of food bundles in retail outlets, particularly whether product bundles at farmers’ market venues could help increase market profits. It may not be feasible for large grocery stores to bundle multiple differing fresh produce items together. However, it may be possible to bundle multiple items of the same produce item or to bundle a fresh produce item with a more shelf-stable food. If bundling is useful in moving more perishable produce items, food bundles may even help reduce food waste and produce shrinkage at the store level. Future studies could examine the influence of food bundles on the rates of in-store produce shrinkage. As demand for online grocery shopping increases, offering food bundles in an online platform could be a relatively easy and cost-efficient marketing strategy to move more fresh produce. Retailers could also display food bundles close to store entrances in order to grab the attention of consumers who may be shopping under cognitive load or who may dislike food shopping. Direct-to-consumer purchasing venues, such as farmers’ markets and co-ops, may also find food bundles a helpful marketing tool to increase consumer sales, particularly for fresh produce.

## Figures and Tables

**Table 1:** Preassembled food bundles featured in the grocery shopping experiment.

Food Bundle	Food Items
Bundle 1	2 Gala Apples;
	3 Bananas;
	1 Bag Frozen Sweet Corn (12 oz);
	1 Can Green Beans (14 oz);
	1 Can Tomatoes (14.5 oz).
Bundle 2	2 Navel Oranges;
	1 Bag Baby Carrots (16 oz);
	1 Bag Frozen Broccoli Cuts (12 oz);
	1 Can Tomatoes (14.5 oz);
	1 Can Bartlett Pears (15 oz).
Bundle 3	3 Bananas;
	2 Cucumbers;
	1 Bag Baby Carrots (16 oz);
	1 Can Tomatoes (14.5 oz);
	1 Can Kidney Beans (15.5 oz).
Bundle 4	2 Navel Oranges;
	3 Bananas;
	1 Bag Frozen Sweet Corn (12 oz);
	1 Can Green Beans (14 oz);
	1 Box Frozen French Fries (4.75 oz).
Bundle 5	2 Cucumbers;
	1 Bag Baby Carrots (16 oz);
	1 Bag Frozen Broccoli Cuts (12 oz);
	1 Box White Rice (16 oz);
	1 Box Elbow Macaroni (16 oz).
Bundle 6	3 Bananas;
	2 Navel Oranges;
	1 Can Bartlett Pears (15 oz);
	1 Container Cheerios (1.3 oz);
	1 Bottle 2% Milk (8 oz).

**Table 2:** Treatments, Grocery Shopping Experiment.

Treatment	Grocery Display	Cognitive Load Condition	*N* ^1^ (Presented First) ^2^	Number of Participants
T1-NL	Bundles	No Load	126	
			(60)	
T1-HL	Bundles	High Load	126	126
			(66)	
T2-NL	Discount Bundles	No Load	124	
			(58)	124
T2-HL	Discount Bundles	High Load	124	
			(66)	
		Total:	500	250

^1^ The experimental design included both between-subjects and within-subjects components. Participants were first randomly assigned at the individual level to a grocery display (between subjects), and then completed the same food task twice (within subjects): once under no load (NL) and once under high load (HL). ^2^ Within subjects, whether participants completed the NL or HL condition first was also randomized at the individual level. NOTE: All grocery displays featured 30 individual food items in addition to the above-indicated display of bundles.

**Table 3:** Description of Variables.

Variable Name	Description ^1^
*HighCognitiveLoad*	1 if participant was under high cognitive load
*DiscountedBundle*	1 if displayed product bundles were discounted 20%
*LowerNCSscore*	1 if participant scored in the lower half on the Need for Cognition Scale (NCS)
*ComplimentaryItems*	1 if participant indicated they “agree” or “strongly agree” that the items in the bundles went well together
*Hungry*	1 if participant indicated they were “very” or “extremely” hungry
*DoesNotEnjoyFoodShopping*	1 if participant indicated they “disagree” or “strongly disagree” that they enjoy food shopping
*PlannedToPurchaseSoon*	1 if participant had already planned to purchase any of their selected items elsewhere
*FollowingSpecialDiet*	1 if participant had a dietary restriction
*ChildrenUnder18*	1 if children under 18 years in the household
*NonCaucasian*	1 if not Caucasian
*Male*	1 if male
*Age*	In years
*HouseholdIncome10K*	Household income (in USD tens of thousands)

^1^ All except *Age* and *HouseholdIncome10K* are dummy variables where the value is zero otherwise.

**Table 4:** Percentage of Consumers Selecting Food Bundles, by presentation order and treatment.

Treatment ^1^	Presented First, % Selecting:				Presented Second, % Selecting:		
	1 Bundle	2 Bundles	Any Bundles (Std Dev)	1 Bundle	2 Bundles	Any Bundles (Std Dev)	Aggregate Any Bundles (Std Dev)
T1-NL (Bundles–No Load)	36.67	8.33	45.0 (50.17)	45.45	3.03	48.48 (47.92)	46.83 (46.07)
T1-HL (Bundles–High Load)	37.88	28.79	66.67 (47.50)	43.33	31.67	75.0 (48.09)	70.63 (42.57)
T2-NL (Discount Bundles–No Load)	25.86	55.17	81.03 (39.55)	22.73	53.03	75.76 (47.44)	78.23 (45.30)
T2-HL (Discount Bundles–High Load)	19.70	36.36	56.06 (50.01)	29.31	34.48	63.79 (47.67)	59.68 (46.59)

^1^ No order effects (significant differences) observed within each treatment, using non-parametric Wilcoxon signed-rank tests for matched pairs. NOTE: This table presents the percentage of participants that selected either 1, 2, or any food bundles during their food-shopping task, by presentation order and treatment. Standard deviations in parentheses.

**Table 5:** Comparison Statistics, by Treatment, for the Percentage of Consumers Selecting Any Food Bundle.

Comparison of Treatments	Avg. % Difference Between Treatments (Std Dev)	*p*-Value ^1^
**Within participants ^2^:**	23.81 (23.40)	<0.001 ***
T1-HL (Bundles–High Load) over T1-NL (Bundles–No Load)		
T2-NL (Discount Bundles–No Load) over T2-HL (Discounted Bundles–High Load)	18.55 (18.41)	0.002 ***
**Between participants ^3^:**		
T1-HL (Bundles–High Load) over T2-HL (Discounted Bundles–High Load)	10.96 (12.29)	0.070 *
T2-NL (Discounted Bundles–No Load) over T1-NL (Bundles–No Load)	31.40 (28.44)	<0.001 ***
T1-HL (Bundles–High Load) over T2-NL (Discounted Bundles–No Load)	−7.59 (7.15)	0.170
T2-HL (Discounted Bundles–High Load) over T1-NL (Bundles–No Load)	12.85 (13.04)	0.031 **

^1^ *p*-values in bold are significant at the 10% *, 5% **, and 1% *** level, respectively. ^2^ *p*-Values for these comparisons were obtained from non-parametric Wilcoxon signed-rank tests for matched pairs. ^3^ *p*-Values for these comparisons are from non-parametric Wilcoxon rank-sum two-sample tests. NOTE: This table presents the average difference in the percentage of consumers selecting 1 or more food bundle(s) during the food-shopping task. Pairwise comparisons are conducted between treatments. Standard deviations in parentheses.

**Table 6:** Random Effects Binary Logit Model and Odds Ratios, Consumer Purchased Any Food Bundle in the Grocery Shopping Experiment.

Variable	Estimated Coefficient (Robust Se)	Odds Ratio (Robust Se)	z-Statistic	*p*-Value ^1^	
*HighCognitiveLoad*	1.380 (0.319)	3.977 (1.268)	4.33	<0.001 ***	
*DiscountedBundle*	1.811 (0.414)	6.118 (2.532)	4.38	<0.001 ***	
*LowerNCSscore*	1.303 (0.558)	3.679 (2.054)	2.33	0.020 **	
*ComplimentaryItems*	0.620 (0.173)	1.858 (0.322)	3.58	<0.001 ***	
*Hungry*	0.731 (0.318)	2.078 (0.660)	2.30	0.021 **	
*DoesNotEnjoyFoodShopping*	1.137 (0.432)	3.116 (1.345)	2.63	0.008 ***	
*PlannedToPurchaseSoon*	−0.883 (0.447)	0.414 (0.185)	–1.98	0.048 **	
*FollowingSpecialDiet*	−1.111 (0.475)	0.329 (0.156)	–2.34	0.019 **	
*ChildrenUnder18*	−0.277 (0.509)	0.758 (0.386)	–0.54	0.587	
*NonCaucasian*	0.243 (0.371)	1.275 (0.474)	0.66	0.512	
*Male*	0.011 (0.013)	1.012 (0.013)	0.93	0.355	
*Age*	0.208 (0.314)	1.232 (0.387)	0.66	0.508	
*HouseholdIncome10K*	−0.021 (0.028)	0.979 (0.027)	–0.74	0.462	
HighCognitiveLoad*DiscountedBundle	−2.792 (0.628)	0.061 (0.038)	–4.45	<0.001 ***	
HighCognitiveLoad*LowerNCSscore	0.581 (0.223)	1.787 (0.399)	2.60	0.009 ***	
HighCognitiveLoad*Hungry	0.582 (0.233)	1.790 (0.417)	2.50	0.012 **	
DoesNotEnjoyFoodShopping*Male	0.537 (0.289)	1.711 (0.494)	1.86	0.063 *	
Constant	−0.772 (2.150)	0.462 (0.994)	−0.36	0.720	
Log pseudolikelihood	−197.6902				
Wald chi-square (17)	53.79				
Prob > chi-square	<0.001				
*N* Observations	500				
*N* Groups	250				

^1^ *p*-values in bold are significant at the 10% *, 5% **, and 1% *** level, respectively. Dependent variable is 1 if consumer purchased any food bundle and 0 otherwise.

## Data Availability

The data presented in this study are available on request from the corresponding author. The data are not publicly available due to privacy and ethical reasons.

## Supplementary Materials

The following supporting information can be downloaded at: https://www.mdpi.com/article/10.3390/foods11070973/s1, Table S1: Order of Grocery Shopping Experiment Tasks.
