# Supplementary material for: Consumer Preference for Food Bundles under Cognitive Load: A Grocery Shopping Experiment
Source: Foods. 2022 Mar 27;11(7):973. doi: 10.3390/foods11070973 (PMC8997493; doi:10.3390/foods11070973)
Supplement: Supplementary file 1 [file foods-11-00973-s001.zip › foods-1619753-supplementary.pdf]

Table S1. Order of Grocery Shopping Experiment Tasks

Task	Sub-Task
1. Obtain informed consent	
2. Review session instructions	
3. Practice tasks:	i. Food shopping practice<br>ii. 7-Digit number memorization and recall practice[1]<br>iii. Arithmetic practice
4. View all products in grocery display	
5. Food shopping tasks[2]	i. Food Shopping Section (2 tasks):[3]<br>a. Shop 1 of 2 grocery displays under cognitive load condition<br>b. 5-minute break<br>c. Shop same grocery display, under different cognitive load condition
6. 5-Minute break	
7. Eight arithmetic tasks[4]:	i. Section A (4 tasks)[5]:<br>Multiply m_1 x m_2, where integer $m_1 \sim U$ (13…19) and integer $m_2 \sim U$ (5….9)<br>ii. 5-minute break<br>iii. Section B (4 tasks)[6]:<br>Multiply m_1 x m_2, where integer $m_1 \sim U$ (13…19) and integer $m_2 \sim U$ (5….9) concurrently with a memorization task (7-digit number recall at the end of each task)
8. Post-experiment questionnaire	
9. Receive study incentives:	i. $5 USDs show-up payment<br>ii. Receive food shopping task items (valued at $10 USDs)<br>1 of 2 tasks randomly selected<br>iii. Memorization task payment ($0-$5 USDs)<br>1 of 7 tasks randomly selected; receive additional $5 USDs if correctly recalled 7-digit number, else receive additional $0 USDs<br>iv. Arithmetic Task payment ($0-$3 USDs)

	1 of 8 tasks randomly selected; receive additional $3 USDs if correctly answered problem, else receive additional $0 USDs

[1]Memorization tasks served to induce high cognitive load
[2]Participants randomized to 1 of 2 grocery displays upon arrival and completed 2 shopping tasks: once under No Load and once under High Load; treatments can be viewed in TABLE 1.
[3]Presentation order of cognitive load conditions randomized within section
[4]Arithmetic performance serves as a manipulation check for high cognitive load, following Deck and Jahedi [22].
[5]Order of tasks randomized within section
[6]Half of participants randomly selected to complete Section B first; the 7-digit numbers employed were 4319162; 8568379; 5862413; 2856979
